# Choriocarcinoma in tubal pregnancy: A case report

**DOI:** 10.1002/ccr3.7977

**Published:** 2023-09-27

**Authors:** Fateme Sadat Najib, Samaneh Bahrami, Zahra Shiravani, Seyed Mohammad Amin Alavi

**Affiliations:** ^1^ Department of Obstetrics and Gynecology, Division of Oncology Gynecology, School of Medicine Shiraz University of Medical Sciences Shiraz Iran; ^2^ Infertility Research Center Shiraz University of Medical Sciences Shiraz Iran; ^3^ Department of Obstetrics and Gynecology, Division of Perinatology, Imam Khomeini Hospital Ahvaz Jundishapur University of Medical Sciences Ahvaz Iran; ^4^ Maternal‐Fetal Medicine Research Center Shiraz University of Medical Sciences Shiraz Iran; ^5^ Faculty of Medicine Ahvaz Jundishapur University of Medical Sciences Ahvaz Iran

**Keywords:** chemotherapy, choriocarcinoma, ectopic pregnancy, fallopian tube, human chorionic gonadotropin, salpingectomy

## Abstract

**Key Clinical Message:**

Choriocarcinoma of the fallopian tube is extremely rare and highly susceptible to early metastasis. Clinical manifestations of ectopic pregnancy and choriocarcinoma are the same, and all patients with ectopic pregnancy should be evaluated for choriocarcinoma based on histopathological findings. Adjuvant chemotherapy (after surgery) is the proposed treatment for tubal choriocarcinoma.

**Abstract:**

Choriocarcinoma is a malignant epithelial tumor of the chorionic villi that often manifests after a normal or molar pregnancy. The primary tubal choriocarcinoma associated with ectopic pregnancy is extremely rare and can be misdiagnosed as an ectopic pregnancy since symptoms including vaginal bleeding, amenorrhea, elevated beta‐human chorionic gonadotropin (BHCG) levels, and pelvic pain are shared. A 34‐year‐old G4P3003 woman presented with a one‐week history of vaginal bleeding and right lower abdominal pain, which had intensified a day before admission. Clinical and paraclinical examinations pointed to a ruptured tubal pregnancy; hence, an emergency laparotomy was performed, and a right salpingectomy was carried out on the patient. However, a nonsignificant decline in BHCG level was observed, and histological examination revealed tubal choriocarcinoma; hence, a metastasis workup was carried out, yet no metastasis was detected. Six sessions of chemotherapy consisting of Etoposide, Methotrexate, Dactinomycin, Cyclophosphamide, and Vincristine (EMA‐CO) were administered without complication, in such a way that the BHCG level normalized after three sessions of chemotherapy. Evaluations after 1 year from the completion of chemotherapy revealed that the patient had no subsequent problems. Choriocarcinoma of the fallopian tube is extremely rare and highly susceptible to early metastasis. Clinical manifestations of ectopic pregnancy and choriocarcinoma are the same, and all patients with ectopic pregnancy should be evaluated for choriocarcinoma based on histopathological findings. Metastasis workup should be considered for all individuals with choriocarcinoma. Adjuvant chemotherapy (after surgery) is the proposed treatment for tubal choriocarcinoma.

## Graphical abstract

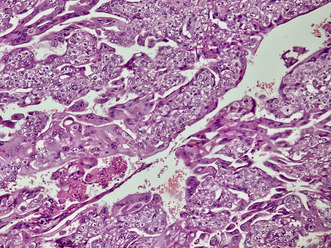

## 1 INTRODUCTION

Choriocarcinoma is the most malignant type of gestational trophoblastic disease (GTD), which is caused by neoplastic alterations in the chorionic villi epithelium and typically arises after a molar pregnancy; however, in rare cases, it can rarely develop after term pregnancy, abortion, or an ectopic pregnancy.^1^, ^2^ Other types of GTD include hydatidiform moles (partial and complete), invasive moles, choriocarcinoma, and placental site trophoblastic tumors.^3^

The origin of choriocarcinoma is usually the uterus and consists of either gestational or non‐gestational types.^1^, ^4^ Tubal choriocarcinoma is extremely rare, and the incidence is reported to be 1.5/1,000,000 births, and accounts for only 4% of all reported cases of choriocarcinoma.^4^, ^5^ Seventy‐six percent of choriocarcinomas develop in ectopic pregnancies and are often accompanied by distant metastases. The onset of choriocarcinomas may range from 5 weeks to 5 years after gestation or even after menopause.^6^ A tubal choriocarcinoma may be misdiagnosed with an ectopic pregnancy since both share similar symptoms, including vaginal bleeding, amenorrhea, elevated beta‐human chorionic gonadotropin (BHCG) levels, and pelvic pain.^7^

Herein, the authors present a rare case of choriocarcinoma in the fallopian tube, for which salpingectomy and chemotherapy were applied as treatment, after which no evidence of recurrence was observed a year after the final chemotherapy session.

## 2 CASE PRESENTATION

A 34‐year‐old G4P3003 (last delivery was 2 years before admission) female presented to the hospital with a week's history of vaginal bleeding and right lower abdominal pain, which had intensified a day before admission. She had regular menstrual periods; however, the last menstrual period was 2 months prior to admission. Her medical history was unremarkable, and she had no history of previous surgery. The vital signs on arrival time were blood pressure: 100/60 mmHg, pulse rate: 98, respiratory rate: 18, and a body temperature of 37°C. Abdominal examination revealed mild tenderness and rebound tenderness in the right lower quadrant. The speculum revealed active bleeding passing through the external orifice of the cervix, while the bimanual pelvic examination revealed cervical motion tenderness and tenderness in the right adnexa with gentle palpation; however, no adnexal mass was detected in the bimanual pelvic examination.

**FIGURE 1:**
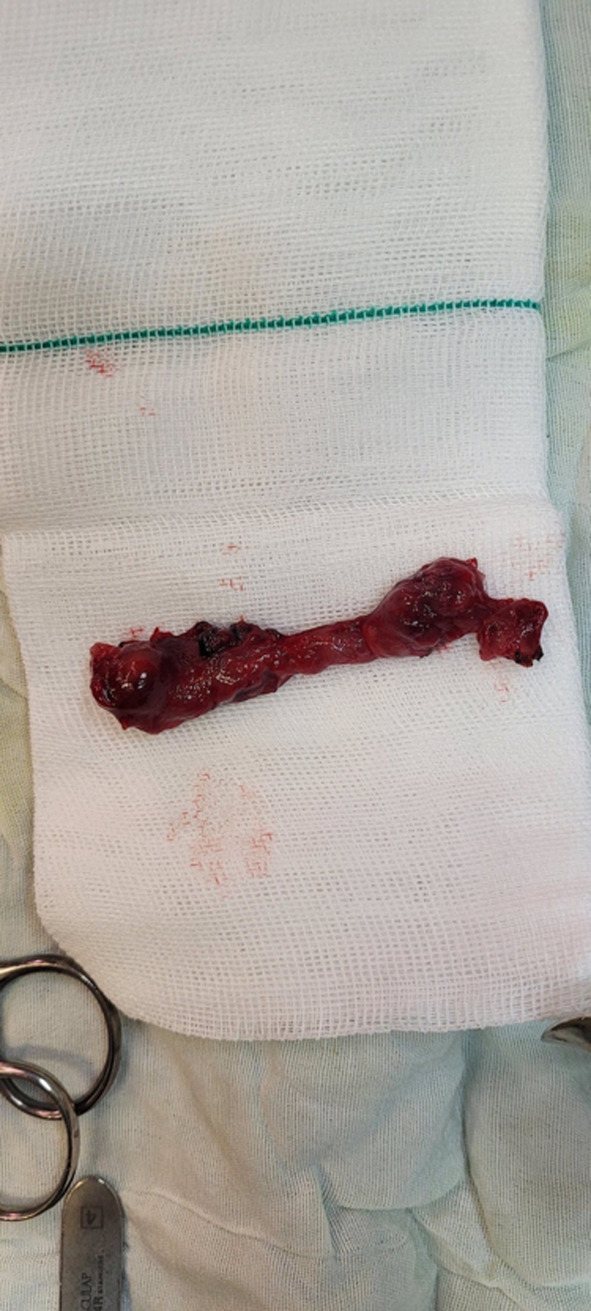
Right salpingectomy due to ruptured right tubal pregnancy with active bleeding.

Laboratory analysis was requested, and the results showed serum BHCG was 14,000 IU/L (0–5 IU/L) and hemoglobin was 8 g/dL (12.1–15.1 g/dL in healthy females). A transvaginal ultrasound was carried out, revealing a 7‐week gestational sac in the right adnexa and moderate free fluid with an internal echo in the pelvic cavity; however, no fetal pole was observed. Clinical and paraclinical analysis pointed to a ruptured tubal pregnancy, and an emergency laparotomy was performed. The intraoperative assessment revealed a 500‐cc clot, fresh blood in the pelvic cavity, and a normal‐appearing uterus and left adnexal structure. The patient underwent a right salpingectomy as a result of a ruptured right tubal pregnancy with active bleeding (Figure 1). One unit of packed red blood cells was transfused during the surgery.

**FIGURE 2:**
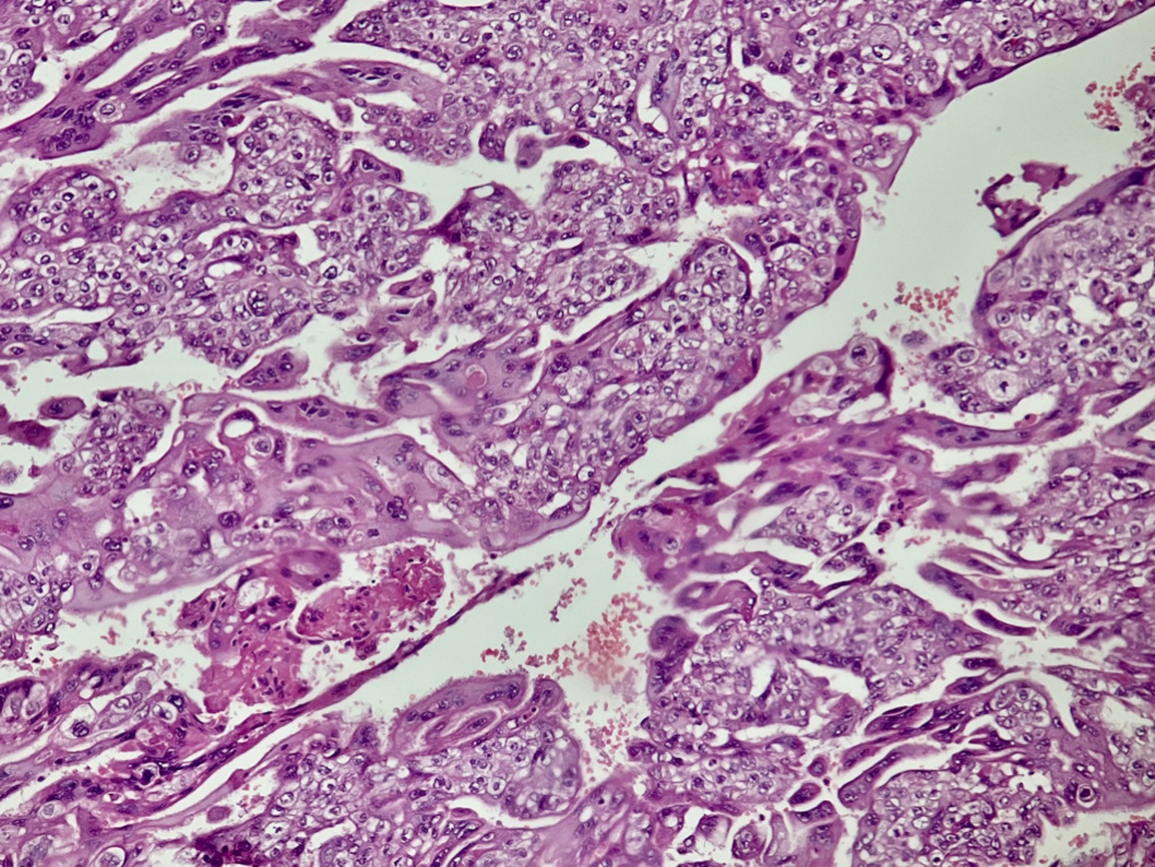
Surgical specimen demonstrating sheets of atypical syncytiotrophoblast and cytotrophoblast and absence of chorionic villi highly suggestive of choriocarcinoma (hematoxylin and eosin stain [100×]).

**FIGURE 3:**
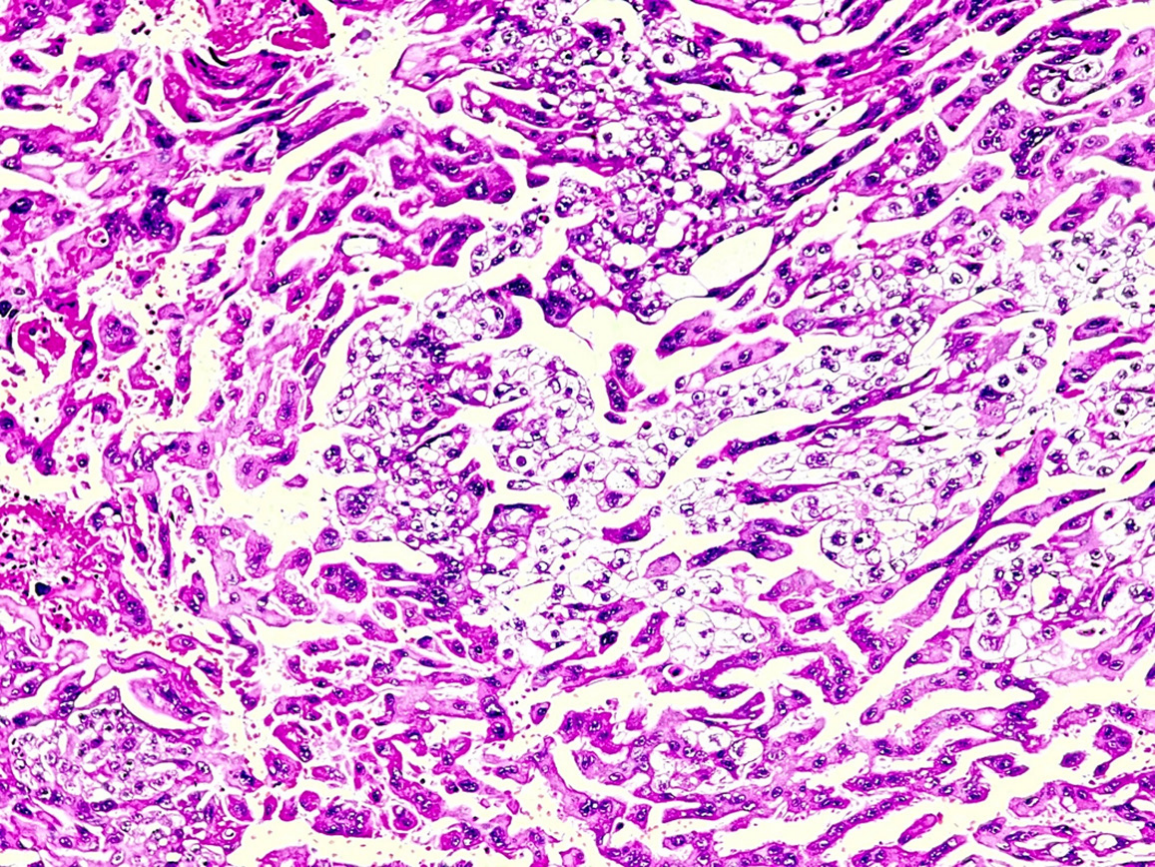
Surgical specimen demonstrating sheets of atypical syncytiotrophoblast and cytotrophoblast and absence of chorionic villi highly suggestive of choriocarcinoma (hematoxylin and eosin stain [200×]).

After 1 day, the patient was discharged from the hospital in good condition. However, after 2 weeks during the follow‐up, the BHCG level did not decrease significantly (BHCG: 12000). Histological examination indicated tubal choriocarcinoma measuring 2 × 1.5 cm with the presence of syncytiotrophoblasts and cytotrophoblast cells engaging the ampulla region of the tubal wall (Figures 2 and 3). The initial pathologic report was also reviewed by a second pathologist, who confirmed the diagnosis.

Chest radiography and abdominopelvic magnetic resonance imaging (MRI) were requested for metastasis workup, all of which were normal. The patient was classified as stage II: 2 based on the International Federation of Gynecology and Obstetrics (FIGO), and the prognostic factor was six as per the World Health Organization (WHO) classification.^8^

Six sessions of chemotherapy consisting of etoposide, methotrexate, dactinomycin, cyclophosphamide, and vincristine (EMA‐CO) were completed without complications. The EMA‐CO was divided into two regimens, which alternated each week. Regimen one consisted of etoposide 100 mg/m2 intravenous infusion over 30 min, actinomycin D 0.5 mg intravenous bolus, and methotrexate 100 mg/m2 intravenous bolus for the first day, and etoposide 100 mg/m2 intravenous infusion over 30 min, actinomycin D 0.5 mg intravenous bolus, and folinic acid rescue 15 mg intramuscularly every 12 h for four doses (starting 24 h after initiation of methotrexate). Regimen two consisted of vincristine 1 mg/m2 intravenous bolus and cyclophosphamide 600 mg/m2 intravenous infusion over 30 min.^8^

The patient was monitored via serial BHCG level, and it was observed that after three chemotherapy sessions, the BHCG level normalized. The patient was advised to use contraceptives during sexual activity. After the chemotherapy sessions were completed, several follow‐ups were carried out at various periods over a year, revealing that the patient had no subsequent problems.

## 3 DISCUSSION

Primary choriocarcinoma of the fallopian tube is extremely rare, with around 100 documented cases since 1981.^9^ The disease often occurs between ages 16 and 56, with an average set at 33 years of age.^4^

According to the literature, the differentiation between tubal choriocarcinoma and tubal ectopic pregnancy is that the former patients have unusually high BHCG levels shortly after amenorrhea; however, the latter patients have BHCG levels that seldom reach 10,000 mIU/mL.^4^ This differentiation is essential in order to properly dissect and subsequently examine the removed lesions during surgical procedures. When there is no visible presence of villous structures in the excised lesions, it is highly recommended to conduct an intraoperative frozen section biopsy to determine whether the lesions are related to ectopic pregnancy or tubal choriocarcinoma.^4^ The gold standard diagnosis for choriocarcinoma is a histopathology evaluation in which columns of trophoblastic cells in the absence of villous structures are manifest, compounded by the invasion of blood vessels and muscle tissue, accompanied by extensive necrosis and hemorrhage.^7^

The differential diagnosis of tubal choriocarcinoma encompasses other conditions, such as ectopic pregnancy without trophoblastic illness, metastatic gestational choriocarcinoma, primary non‐gestational choriocarcinoma, placental site nodule, placental site trophoblastic tumor, and epithelioid trophoblastic tumor.^10^ It is of note that immunohistochemistry analysis using BHCG, Ki 67, cytokeratin, placental alkaline phosphates (PLAP), and CD30 can help physicians in differential diagnosis^11^; however, in the current study, immunohistochemistry was not utilized.

Early metastasis is the most important aspect of choriocarcinoma.^4^ The most common site of metastasis is the lungs (80%), followed by the vagina (30%), pelvis (20%), liver (10%), and brain (10%).^12^ Early diagnosis enhances patients' prognosis because metastatic hemorrhage is a leading cause of mortality; what's more, the diagnosis should rule out all other forms of pregnancy, including normal intrauterine pregnancies.^6^

From a surgical point of view, salpingectomy is recommended for young patients who seek to retain their fertility. Adnexectomy and total hysterectomy with bilateral adnexectomy have been described in the literature and are recommended for older women and/or in the absence of a desire for pregnancy.^13^

The use of chemotherapy has completely transformed the prognosis of choriocarcinomas since choriocarcinomas are highly susceptible to chemotherapy, in as such that chemotherapy significantly increases the possibility of a cure even in the presence of multiple metastases.^14^ The literature emphasizes that patient survival rates have increased from 19% when the treatment was only surgical to more than 90% since the use of chemotherapy.^13^ Patients with choriocarcinoma are classified as high‐risk based on the FIGO and are usually associated with an elevated risk of single‐agent chemotherapy; thus, the most common chemotherapy regimen for such patients is EMA‐CO.^8^ The reporting of staging and risk assessment, which are currently utilized by FIGO and WHO as fundamental components of GTN therapy and prognosis. However, the aforementioned scores were not often reported in previous studies, and the EMA‐CO chemotherapy regimen was mainly administered to patients with choriocarcinoma.^15^

It is recommended that women who have undergone chemotherapy for tubal choriocarcinoma should be provided with reliable contraceptive methods for a minimum duration of 1 year following the treatment.^7^

Lifelong BHCG levels and imaging examinations are crucial for individuals diagnosed with tubal choriocarcinoma, as no definitive guideline specifies the appropriate cessation point for follow‐up.^4^

The presented case was a 34‐year‐old woman whose symptoms indicated tubal ectopic pregnancy. The onset age of the current patient and symptoms were similar to previous studies. A salpingectomy was carried out for the patient, and the histopathological findings demonstrated tubal choriocarcinoma. Fortunately, the results of the metastasis workup were negative. In addition, the patient underwent six sessions of EMA‐CO chemotherapy, and no subsequent problems were observed after 1 year.

## 4 CONCLUSION

Choriocarcinoma of the fallopian tube is extremely rare and highly susceptible to early metastasis. Since ectopic pregnancy and choriocarcinoma have similar clinical manifestations, all patients with ectopic pregnancy should be evaluated for choriocarcinoma based on histopathological findings. Moreover, metastasis workup should be considered for all individuals with choriocarcinoma. Adjuvant chemotherapy (after surgery) is highly recommended as a treatment for tubal choriocarcinoma.

## AUTHOR CONTRIBUTIONS

**Fateme Sadat Najib:** Conceptualization; writing – original draft; writing – review and editing. **Samaneh Bahrami:** Conceptualization; writing – original draft; writing – review and editing. **Zahra Shiravani:** Conceptualization; writing – original draft; writing – review and editing. **Seyed Mohammad Amin Alavi:** Conceptualization; writing – original draft; writing – review and editing.

## FUNDING INFORMATION

No sources of funding were declared for this study.

## CONFLICT OF INTEREST STATEMENT

The authors have no conflict of interest to declare.

## ETHICS STATEMENT

Permission from the ethics committee was not required for case reports at the institution where the research was carried out.

## CONSENT

Written informed consent was obtained from the patient to publish this report in accordance with the journal's patient consent policy.

## Data Availability

The data supporting the findings of this study are available upon request from the corresponding author.
